# The Immunomodulatory and Antimicrobial Properties of the Vertebrate Ribonuclease A Superfamily

**DOI:** 10.3390/vaccines6040076

**Published:** 2018-11-20

**Authors:** Laura Schwartz, Ariel Cohen, Jason Thomas, John David Spencer

**Affiliations:** 1 Center for Clinical and Translational Research, The Research Institute at Nationwide Children’s, Columbus, OH 43205, USA; laura.schwartz@nationwidechildrens.org (L.S.); acohen4@wellesley.edu (A.C.); 2 The University of Toledo College of Medicine and Life Sciences, Toledo, OH 43614, USA; 3 Division of Nephrology, Department of Pediatrics, Nationwide Children’s, Columbus, OH 43205, USA; jason.thomas@nationwidechildrens.org

**Keywords:** ribonuclease A superfamily, ribonucleases, innate immunity, infection, inflammation

## Abstract

The Ribonuclease A Superfamily is composed of cationic peptides that are secreted by immune cells and epithelial tissues. Although their physiological roles are unclear, several members of the vertebrate Ribonuclease A Superfamily demonstrate antimicrobial and immune modulation activities. The objective of this review is to provide an overview of the published literature on the Ribonuclease A Superfamily with an emphasis on each peptide’s regulation, antimicrobial properties, and immunomodulatory functions. As additional insights emerge regarding the mechanisms in which these ribonucleases eradicate invading pathogens and modulate immune function, these ribonucleases may have the potential to be developed as a novel class of therapeutics for some human diseases.

## 1. Introduction

**Figure 1:**
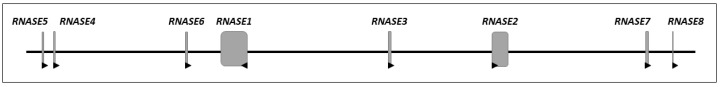
Localization and orientation of human RNase A Superfamily genes on chromosome 14q11.2.

The Ribonuclease A (RNase A) Superfamily is a vertebrate-specific gene family that encodes several peptides found in a range of mammals. In humans, the RNase A Superfamily was originally found to contain eight genes located on chromosome 14q11.2 (Figure 1). These genes encode eight cationic polypeptides (approximately 15 kDa in size), including pancreatic RNase (also known as RNase 1), eosinophil-derived neurotoxin (EDN/RNase 2), eosinophil cationic protein (ECP/RNase 3), RNase 4, angiogenin (RNase 5), RNase 6 (k6), RNase 7, and RNase 8.

**Figure 2:**
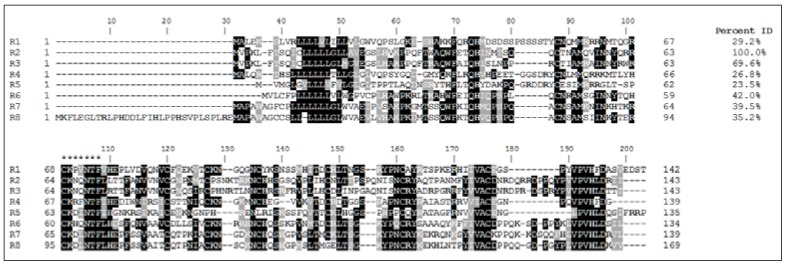
Ribonuclease A Superfamily amino acid sequences and peptide structures. The single letter amino acid sequence for full-length RNase 1 (R1) to RNase 8 (R8) aligned using Clustal Omega is shown. Percent identities of the peptides are shown to the right. Amino acids shaded darker indicate higher degree of conservation among peptides. Asterisks (bottom left) highlight the conserved CKXXNTF catalytic motif. Cationic amino acids are indicated in boldface type.

Following sequencing of the human genome, five additional ribonucleases were identified in close proximity to RNases 1–8 on chromosome 14: RNases 9–13 [1,2]. These RNases were termed “noncanonical” due to the absence of a catalytic domain and consequent lack of ribonuclease activity. Among the common elements in the canonical RNases, each gene encodes a 20–28 amino acid signal peptide and a mature peptide of about 130 amino acids. Seven of the eight genes encode eight cysteines, forming four disulfide bonds in the secreted and appropriately folded peptides. The exception is RNase 5, whose gene encodes six cysteines and subsequently a peptide with three disulfide bonds. Each gene encodes a conserved signature catalytic motif (CKXXNTF). While the encoded canonical peptides are catalytically active with varying degrees of activity against standard polymeric RNA substrates, the catalytic activity may be unrelated to the peptide’s antimicrobial activities. Outside of the catalytic motif, the RNase A peptides display remarkable sequence diversity with sequence identities ranging from 20 to nearly 70% (Figure 2). This sequence diversity may play a role in promoting the functions of each peptide. It is of interest to note that denatured RNases retain their antimicrobial function despite a loss of catalytic capability, bypassing the structure-to-function paradigm [1,2,3,4].

## 2. Evolution of the Ribonuclease A Superfamily

**Figure 3:**
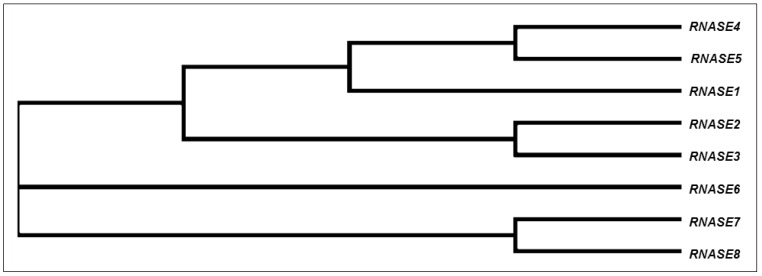
Phylogenetic tree showing the relationships among the human Ribonuclease A Superfamily gene lineages. Genetic sequences were aligned using Clustal Omega, then a neighbor-joining phylogenetic tree was generated using Simple Phylogeny.

The RNase A Superfamily is believed to be the only enzyme family that is vertebrate-specific. Although non-vertebrate organisms possess RNases, they are non-homologous to vertebrate RNase A. Phylogenic studies indicate that the RNase A superfamily originated from an RNase 5-like gene and expanded during mammalian evolution [1,5]. Among mammals, RNases have been studied in primates, rodents, and ruminants. Analysis of RNase sequences between species has revealed that the RNase A Superfamily is one of the most rapidly evolving mammalian families, resulting in variable numbers of genes in different species [1]. The phylogenetic tree in Figure 3 depicts the relationships among the human RNase A Superfamily genes. This neighbor-joining phylogenetic tree is generated from the multiple sequence alignment of the RNase A Superfamily genes and aims to represent the evolutionary relationship between sequences [1,6,7]. 

Rapid gene duplication has been described among other immune-related gene families and proteins, including the major immunohistocompatibility complex, immunoglobulins, and T cell receptors [7]. Since distinct lineages of this superfamily have antimicrobial function (as outlined below), some suggest that this superfamily primarily functions in vertebrate host defense [1,4,5]. The following sections outline the emerging roles these RNase A Superfamily members may play in the inflammatory response and host defense. 

## 3. The Antimicrobial and Immunomodulatory Properties of the Ribonuclease A Superfamily

RNase A Superfamily peptides are produced by circulating immune cells and microbe-challenged epithelial surfaces—including keratinocytes, respiratory tract epithelium, and urothelial cells in the genitourinary tract [8,9]. Thus, they are ideally situated to facilitate immunoprotective responses by directly killing invading pathogens or activating inflammatory responses. Like other antimicrobial peptides and proteins, RNase A peptides have characteristics that allow them to interact with invading pathogens. Similar to defensins and cathelicidin, RNase A peptides have a net positive charge, which allows them to bind microbial membranes. In addition, they can form amphipathic structures in hydrophobic microenvironments, allowing them to penetrate the bacterial phospholipid bilayer [5,10]. Elegant studies by Torrent and colleagues have shown that the antimicrobial activity of RNase A peptides is largely confined to the N-terminus, which may have been selected by evolution to provide a host-defense function [11]. Published literature suggests that RNases have cytotoxic, anti-helminth, antibacterial, antiviral, and antifungal activity [4,12,13,14,15,16,17,18].

**Figure 4:**
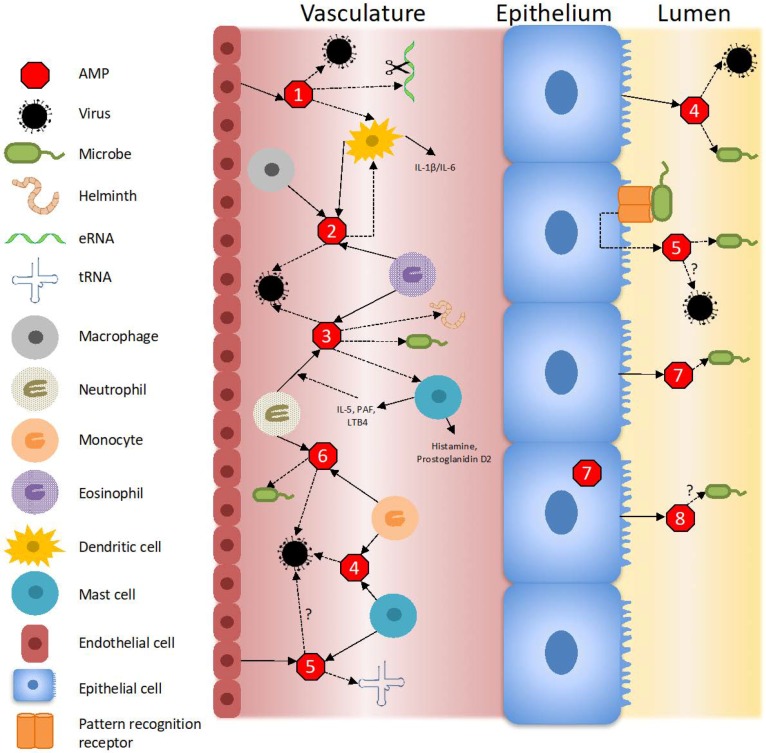
Innate immune mechanisms of the Ribonuclease A Superfamily. RNases are indicated by red octagons with the number inside corresponding to the specific RNase. RNase 1 is secreted by endothelial cells and degrades extracellular RNA (eRNA). RNase 1 also activates dendritic cells, which secrete inflammatory factors such as IL-1β and IL-6. Antiviral activity by RNase 1 has also been reported. RNase 2 is primarily secreted by eosinophils, but also dendritic cells and macrophages. RNase 2 can then signal back to dendritic cells, activating them and inducing their release of inflammatory factors. RNase 2 has antiviral activity. RNase 3 is secreted from eosinophils, as well as neutrophils and mast cells. A positive feedback mechanism occurs when mast cells release inflammatory factors and induce neutrophil production of RNase 3. RNase 3 exhibits antiviral and antimicrobial activity. RNase 4 is released from monocytes and mast cells, as well as epithelial cells, and exhibits antiviral and antimicrobial activity. RNase 5 is secreted from endothelial cells and mast cells and targets noncoding RNA. RNase 5 is also released from epithelial cells in response to pattern recognition receptor activation, where it may demonstrate antimicrobial action. RNase 6 is released from circulating neutrophils and monocytes and possesses antimicrobial and antiviral activity. RNase 7 and 8 are secreted from epithelial cells. RNase 7 protects from microbial infection while the antimicrobial function of RNase 8 is less defined.

To date, the diverse physiological functions of the RNase A Superfamily members are still being defined. In addition to their direct anti-pathogen functions, RNase A peptides modulate inflammatory responses via different mechanisms. They can inhibit the expression of pro-inflammatory cytokines and chemokines. Moreover, they can act as alarmins, chemoattractants, opsonins, and activate immune cells. Additionally, they promote the clearance of extracellular RNA (eRNA). Evidence also suggests that RNases contribute to degradation of dietary RNAs, angiogenesis, neurotoxicity, and immunomodulation [3,4,8,19,20]. Specifically, they have been shown to play roles in inflammatory skin diseases, oral inflammation, inflammatory neuronal disease, and chronic respiratory tract diseases. While many RNases are acutely induced during infection, they are often downregulated after extended periods of infection. In these later stages of infection, RNases may contribute to tissue repair and remodeling. While participating in the tissue healing process, the RNases can target and remove the host-damaged cells (Figure 4) [4,8,21]. The innate immune functions of the RNase A Superfamily members are summarized in Table 1 and reviewed in the following sections.

## 4. Ribonuclease 1

Ribonuclease 1 (or pancreatic ribonuclease), initially isolated from bovine pancreas, is the archetype of the RNase A superfamily and the best characterized ribonuclease. Since its discovery in the 1950s, it has served as a model peptide in studies investigating protein folding, disulfide bond formation, protein spectroscopy, crystallography, and protein dynamics. In humans, the analog of bovine RNase 1 has been shown to have fairly ubiquitous expression, including organs like pancreas, brain, kidney and spleen, as well as endothelial and genitourinary tissues [5,10]. RNase 1 expression has been shown to be regulated at the epigenetic level through chromatin acetylation [67]. The protein is known to undergo post-translational modification, and purified samples from different organs and tissues show variable patterns of glycosylation. RNase 1 has been shown to play essential roles in digestion, vascular homeostasis, and serum viscosity [10,26,67] 

Recent focus has shifted to the protective role of RNase 1 in mitigating inflammation at sites of tissue damage, vascular disease, or cancer by degrading eRNA [20,21,22]. Endothelial cells are a major source of RNase 1, where it is stored in granules termed Weibel–Palade bodies. The release of eRNA from damaged cells as well as cancer cells has been shown to initiate cytokine signaling that is attributed to increased vascular permeability. The ribonuclease activity of RNase 1 has been shown to attenuate this response [68]. Others have shown that RNase 1 protects alveolar epithelium from pneumococcal infiltration [69] and prevents Rheumatoid arthritis-associated fibroblast interaction with cartilage [70]. 

In regards to host defense, RNase 1 is not recognized as a robust antimicrobial or cytotoxin. However, evidence suggests that RNase 1 has antiviral activity. Recombinant RNase 1 peptide and urinary RNase 1 extracts from pregnant women showed activity against human immunodeficiency virus (HIV-1) [18,71]. Additionally, recombinant RNase 1 may affect host anti-pathogen responses by activating human dendritic cells, leading to the production of inflammatory cytokines, chemokines, growth factors, and soluble receptors [24]. While it is not clear how RNase 1 contributes to innate immune functions, further exploration of its host defense and immunomodulatory properties may be warranted.

## 5. The Eosinophilic Ribonucleases—Ribonuclease 2 and 3

Eosinophil granule proteins were among the first proteins in the RNase A Superfamily to demonstrate a role in innate immunity. Eosinophil-derived neurotoxin (EDN or RNase 2) was first described in the 1930s when rabbits developed neurotoxicity after receiving an intracerebral injection of a human lymph node suspension. It was determined that this neurotoxic effect was secondary to a protein isolated from the granules of infiltrating eosinophils, EDN [72]. It remains unclear whether EDN’s catalytic activity is responsible for its neurotoxic effects and/or the physiological benefits of its neurotoxicity. Although EDN expression has been detected in cells besides eosinophils—including monocytes, dendritic cells, basophils, and neutrophils—this protein is best known as an eosinophil constituent that is present in this cell at high concentrations [73]. Evidence suggests that EDN has promise as a biomarker in eosinophil-associated disease states, including asthma exacerbation, cow’s milk allergy, eosinophilic esophagitis, vaccine induced aberrant responses, and respiratory syncytial virus (RSV)-induced bronchiolitis [27,74]. 

In response to inflammatory mediators and cytokines, EDN is released from eosinophilic granules. Eosinophils release EDN in response to invading pathogens via a TLR7-MyD88 pathway [75]. Single-stranded RNA viruses activate the eosinophil’s Toll-like receptor (TLR) 7, causing the eosinophil to express EDN to act on viral RNA and enhance host responses against the infection [21]. In human eosinophil promyeolocitic leukemic cells, *RNASE2* mRNA expression was found to be dependent on GATA-2 transcriptional factor, which has also been implicated in immune cell differentiation, further supporting a role for RNase 2 in immune modulation [76]. In models of airway inflammation and infection, EDN promotes viral clearance [27]. EDN exhibits ribonuclease-dependent antiviral activity against RSV and HIV [16]. 

Evidence suggests that RNase 2 also acts as a chemoattractant, stimulates dendritic cell activation, enhances T helper lymphocyte type 2 (TH2) immune responses, and serves as an endogenous ligand for the pathogen recognition receptor TLR2 [24,27,28]. Given its ability to facilitate antigen recognition, RNase 2 may act as an alarmin [24,27,28]. The expanding roles of RNase 2 in promoting innate immunity and immunomodulation have been reviewed [27]. Moreover, the functions of tissue-resident eosinophils have recently been described [31].

Eosinophilic cationic peptide (ECP or RNase 3) is another RNase A Superfamily member that is found in eosinophilic secretory granules. ECP’s sequence is most similar to EDN and it appears that in humans the two genes arose through a recent gene duplication [77]. Levels of ECP in tissue and peripheral blood correlate with the number of eosinophils present. Besides eosinophils, other leukocyte cells such as neutrophils express ECP. In response to infection and inflammation, circulating immune cells release ECP [78]. Several types of inflammatory stimuli trigger ECP release. Interaction with adhesion molecules, stimulation by leukotriene B4, platelet activating factor, interleukin (IL)-5, immunoglobulins, and complement C3a and C5a have been shown to cause ECP release [33]. Upon its release, ECP can serve as a direct antimicrobial, chemoattractant, or an immunomodulator [33,79].

Since its discovery in 1977, ECP has been used and evaluated as a biomarker to assess activity of various human inflammatory diseases. Several of these diseases are associated with eosinophils and ECP. Most common are diseases associated with atopy and the TH2 phenotype—including asthma, allergic rhinitis, atopic dermatitis, ulcerative colitis, and eosinophilic esophagitis [33,74,79,80]. The following reference provides a comprehensive review of the advantages and pitfalls of ECP as a biomarker in allergic disease [81].

With regard to respiratory tract disease, airway inflammation is closely linked to eosinophil degranulation, which causes local tissue damage. Similarly, inflammatory skin diseases are associated with eosinophil infiltration and deposition of eosinophil proteins. In both tissue types, the detrimental effects of eosinophilic protein tissue deposition is followed by a remodeling process [31]. RNase 3 has remodeling activity that is partly mediated by inducing the expression of epithelial insulin-like growth factor 1 (IGF-1) expression [32]. In addition, RNase 3 can enhance fibroblast chemotaxis to the site of injury to facilitate tissue repair. However, fibroblast recruitment can also lead to fibrosis—as observed with chronic eosinophilic inflammation in lower respiratory tract diseases [34]. 

ECP possesses antibacterial, anti-helminthic, and cytotoxic activities at micromolar concentrations in vitro, suggesting that it plays a role in innate host defense [82,83,84]. The antibacterial properties of RNase 3 are independent of its enzymatic activity, while its antiviral and anti-helminthic activities are dependent on its catalytic function [16]. Lehrer et al. demonstrated that ECP kills both Gram-positive as well as Gram-negative bacteria [13]. Upon binding to bacterial surface polymers (including peptidoglycan or lipopolysaccharide), ECP triggers bacterial agglutination [29,85]. In part, ECP disrupts the bacterial membranes by forming transmembrane pores in the outer lipid bilayers and/or disrupting the membrane through a carpet-like mechanism [29,86,87]. The biological contributions of ECP/RNase 3 to host defense have been reviewed [20,33,88,89].

As many as fifteen murine eosinophil associated ribonucleases (mEars) have been described, all of which are predicted to possess ribonuclease activity based on their structural and catalytic elements [90,91,92,93,94,95,96]. These proteins share only 50% amino acid identity with their human counterparts and exhibit rapid-birth-death, an evolutionary characteristic of other immune response genes and indicator of pathogen-induced evolution [97]. The similarities between human and mouse Ears include their basic nature, low catalytic activity, and diverse biological functions [91,92,95,98]. Recent evidence suggests that mEar 2, mEar 5, mEar 7, and mEar 11 have cytotoxic, antibacterial, and anti-parasitic activity. In addition, mEar 11 acts as a macrophage chemoattractant [98]. Thus, it appears that mEars may have a role in host defense. Ongoing studies are needed to elucidate whether mEars function in physiologically similar ways to human eosinophilic ribonucleases.

## 6. Ribonuclease 4

Among the members of the RNase A Superfamily, RNase 4 is the most conserved gene across the different vertebrate species [2]. Even so, it is one of the least studied RNases. Human RNase 4 is similar in sequence to human RNase 5 and these two genes (*ANG* and *RNASE4*) share a promoter [99]. The same holds true for *Rnase4* and *Rnase5* in mouse [100]. Recombinant RNase 4 peptide is angiogenic, neurogenic, and neuroprotective [35,36]. *RNASE4* mRNA expression has been detected throughout the human body, including tissues like skeletal muscle, pancreas, lung, kidney, placenta, liver, and monocytes, and mast cells [101,102,103]. RNase 4 possesses strong ribonuclease activity and exhibits a preference for uridine at the main base cleavage sites [38,39]. 

Although considerable work has been completed to interpret RNase 4 substrate specificity, a small amount of published literature is available evaluating its biological functions. Some evidence suggests that RNase 4 plays a role in neurodegenerative diseases. A computational gene analysis showed that *RNASE4* transcript expression is markedly upregulated in patients suffering from Huntington’s disease [104]. Similarly, a single nucleotide polymorphism in the *RNASE4* gene has been shown to affect its ribonuclease activity leading to the development of amyotrophic lateral sclerosis (ALS) [36]. Human spinal cord samples from ALS patients showed a significant decrease in *RNASE4* mRNA expression. Treatment with recombinant RNase 4 conferred neuromuscular benefit to a transgenic mouse model of ALS, lending rationale for its consideration as a novel ALS therapeutic by promoting neurogenesis and neuronal survival under stress [35]. The neuroprotective activity of RNase 4 is similar to that seen with RNase 5, which can upregulate Bcl-2 and inhibit nuclear translocation of apoptosis-inducing factor [54].

In regards to host defense, its antimicrobial activity is not well defined. Evidence suggests that purified bovine RNase 4 suppresses the growth of *Candida albicans* and has cytotoxic activity [40,41]. Moreover, RNase 4 was identified, along with RNase 5, among the soluble factors secreted by T cells showing anti-HIV activity [37].

## 7. Ribonuclease 5 (Angiogenin)

Human RNase 5 (angiogenin) is considered the most ancient RNase A Superfamily member, and it shares many structural features with non-mammalian vertebrate RNases. Numerous primate and other mammalian orthologues of human angiogenin have been identified, and like EDN/ECP, appear to evolve from a rapid rate of non-silent substitutions [1,5]. As observed in the eosinophil RNases, the angiogenin lineage has expanded into rodents. Six mouse angiogenin genes (*Ang1*-*6*) and three mouse angiogenin pseudogenes (*mAng-ps1*-*ps3*) have been identified [1,5].

RNase 5 is constitutively expressed in normal blood plasma as well as a wide range of circulating immune cells and epithelial tissues. In contrast to RNase 4, RNase 5’s catalytic activity is relatively weak and shows preferential cleavage of single-stranded RNA as the substrate and the cleavage specificity depends on its secondary structure [105]. Although RNase 5 can bind DNA in vivo, it does not cleave DNA [106].

RNase 5 has been associated with a ride range of physiological processes, including tumorigenesis, reproduction, and tissue regeneration. It has also been implicated in inhibiting HIV-1 replication [37]. As implied by its name, angiogenin is involved with inducing blood vessel growth, which is dependent on its catalytic activity. In a recent study, one group found that administration of angiogenin after lethal irradiation improved animal survival and reduced post-radiation neutropenia [44]. In addition, RNase 5 has been proposed as a viable diagnostic biomarker for cancer, as it has been found to be upregulated in various forms of the disease [42]. The physiological effects of RNase 5/angiogenin have recently been reviewed in detail [43]. 

The role of RNase 5 in immunomodulation has been broadly investigated. Kulka et al. discovered that RNase 5 is produced by mast cells and induced by the endotoxin LPS. The same group found that other TLR ligands were able to induce RNase 5 production, suggesting that mast cell-derived RNase 5 can be secreted in a variety of inflammatory settings [47,48]. In addition to these functions, RNase 5 has been reported to serve as an immunomodulator by inhibiting the degranulation of polymorphonuclear cells at nanomolar concentrations [45]. It has been postulated that RNase 5 regulates inflammatory states by inhibiting TKB1-mediated NF-κB nuclear translocation, thus decreasing levels of TNFα and IL-1β [43,48,107]. RNase 5 has also been implicated in modulating inflammatory responses due to its ability to stimulate leukocytes to produce the pro-inflammatory cytokines IL-6 and TNFα. Shcheglovitova et al. demonstrated that levels of these pro-inflammatory cytokines were increased after the leukocytes were incubated with RNase 5. Furthermore, the degree to which cytokines were produced was proportional to the amount of RNase 5 used [46].

As briefly highlighted above, mutations in *ANG* have been shown to play a role in the development of the progressive neurodegenerative disease, ALS. Treatment of transgenic *SOD1^G93A^* mice with recombinant RNase 5, either alone or in combination with recombinant RNase 4, conferred neuromuscular benefit [35]. RNase 5 can promote cell survival in stress conditions by inducing tiRNA, which inhibits global protein translation, both cap dependent and independent [53].

In the context of antimicrobial activity, human RNase 5 displays toxicity toward *S. pneumoniae* and *C. albicans* [43]. However, conflicting reports also exist [49]. While RNase 5 is secreted by keratinocytes and has been detected in lacrimal secretions, its contribution to skin and ocular defenses has not been extensively investigated [43,48,107]. Further work is warranted to clarify the nature and relative anti-pathogen activities and innate immune functions of RNase 5 in vivo.

## 8. Ribonuclease 6

RNase 6 (k6) is a secreted antimicrobial peptide that was discovered as the human orthologue to bovine RNase K2 [108]. RNase 6’s ribonuclease catalytic activity is relatively weak compared to other canonical RNases–almost 40-fold less than RNase 2/EDN against yeast tRNA [108]. Unique among the Ribonuclease A superfamily members, recent work has identified a secondary catalytic site in RNase 6 which has been suggested to contribute to its enhanced endonuclease specificity in the cleavage of polynucleotide substrates [109]. The published literature primarily focuses on RNase 6’s contributions to host defense [30]. RNase 6 is a myeloid derived protein expressed by monocytes, macrophages, and neutrophils [108,110]. Similarly, murine RNase 6 is detected by invading monocytes and macrophages in the genitourinary tract after *E. coli* challenge [55]. Human and mouse RNase 6 demonstrate bactericidal activity against Gram-positive and Gram-negative pathogens [30,55]. The antimicrobial mechanisms of RNase 6 resemble RNase 3 in that it induces bacterial agglutination and membrane depolarization in Gram-negative bacteria [30]. Bovine RNase 6 is upregulated during pregnancy in CD14+ endometrial macrophages, where it is believed that macrophages are recruited to facilitate the necessary immune changes for survival of fetus and for host defense [56]. As demonstrated with RNases 1, 2, 4, and 5, RNase 6 also inhibits HIV replication and is downregulated in Th17 cells infected with HIV [57].

## 9. Ribonuclease 7

Ribonuclease 7 (RNase 7) was first identified in human skin and has since been localized to other epithelial surfaces including the respiratory and genitourinary tracts [15,59,111]. To date, the only RNase 7 coding sequences that have been characterized are in higher order vertebrates, thus limiting our ability to determine if RNase 7 is undergoing the rapid evolution as observed for ECP and EDN [1,15]. Neither the mouse or the rat genome contains sequences orthologous to RNase 7, which limit our ability to assess its biological activity in vivo [1,2,5]. 

RNase 7 is constitutively expressed and secreted by mucosal tissues at high concentrations, yet its expression can be induced in response to growth factors, cytokines, UV light, and bacterial products. [15,112,113,114,115,116,117]. The molecular mechanisms in which these heterogeneous stimuli induce RNase 7 expression are not well characterized, however published literature suggests that Toll-Like Receptor (TLR)-mediated pathways as well as mitogen-activated protein kinase (MAPK) and phosphatidylinositol 3-kinase/AKT (PI3K/AKT) signaling may regulate RNase 7 expression [112,114,118,119].

Cutaneous RNase 7 expression is upregulated in chronic inflammatory skin diseases such as atopic dermatitis and psoriasis [120,121]. Both of these conditions are characterized by the infiltration of activated T cells into the epidermal compartment which release TH2 cytokines. Recent data shows that RNase 7 can selectively suppress TH2 cytokine production by reducing GATA3 activation [62,120,121]. It has further been suggested that RNase 7 functions as an immunomodulatory molecule promoting inflammation and chemotaxis in acne and cutaneous wound healing [122]. Finally, emerging evidence indicates that RNase 7 acts as an alarmin by converting self-DNA released by dying host cells into a danger signal that rapidly activates interferon genes and anti-microbial immune responses. The findings presented in this study suggest that RNase 7 plays a role immunomodulation to facilitate microbial clearance or drive autoimmunity in chronic inflammatory skin conditions by permitting recognition of self-DNA by plasma dendritic cells [58].

In addition to these immunomodulatory effects, RNase 7 is probably the best example of an RNase that can shield the host from pathogens. RNase 7 possesses broad-spectrum antimicrobial activity against Gram-positive and Gram-negative bacteria as well as yeast [15,60,123]. Unlike other RNases, it has limited antiviral activity [124]. Similar to RNase 3, mutation and truncation studies show that RNase 7’s antimicrobial activity is independent of its ribonuclease activity, and instead is dependent on its ability to disrupt the bacterial cell wall [15,29,61,87,124]. Its ability to disrupt bacterial membranes is conserved at the N-terminus [11,125]. Site-directed mutagenesis studies have identified a cluster of lysine residues at the flexible coil near RNase 7’s N-terminus that are critical for its bactericidal activity [60]. RNase 7’s structure, function, and antimicrobial activity have been recently reviewed [8,126].

## 10. Ribonuclease 8

RNase 8 is the last canonical RNase A Superfamily member to be identified and its antimicrobial activity is not well characterized. The published literature primarily describes its antimicrobial activity. It was initially described as non-microbicidal [64]. However, later studies show that recombinant RNase 8 peptide exhibits sub-micromolar toxicity to a variety of pathogens, including Gram-positive and Gram-negative bacteria as well as yeast [17]. While maintaining high sequence identity with other canonical Ribonuclease A family members, especially RNase 7, RNase 8 is unique in that glycine has replaced a cysteine residue that is conserved in all other RNase A family members. In addition, another cysteine residue has appeared downstream, suggesting a new possible disulfide bridge site. Mass spectrometry analysis of RNase 8 supports this hypothesis [64]. Finally, a unique extension at the N-terminus may indicate that this peptide is not undergoing secretion shared by the other canonical RNases [21,127].

Northern blot analysis initially detected *RNASE8* mRNA in human placenta and ensuing studies show that it is also expressed in adult spleen, lung, and testis [64,127]. RNase 8’s antimicrobial properties and high levels of expression in the placenta suggest it may play a role in the innate defenses of pregnant women. This implies an additional role for the RNase A Superfamily in host defense—i.e., as defense system between the mother and her fetus since pathogens can cross the placenta to the fetus [10]. However, given the conflicting reports regarding the peptides antimicrobial activity, additional studies are needed to define this peptide’s function.

## 11. The Non-Canonical Ribonucleases—Ribonuclease 9–13

The RNase A Superfamily was expanded with the discovery of several mammalian members. RNases 9-13 are secreted, inactive ribonucleases and are termed non-canonical ribonucleases due to their lack of ribonuclease catalytic activity [1]. These non-canonical RNases are expressed in primates as well as other higher-order vertebrates, including rodents. The primary structure of these peptides resembles ancestral RNases, sharing the three most conserved disulfide bonds and a secretion peptide, but not the N-terminal region of the mature proteins [1,2,21].

RNases 9–13 are expressed predominantly in the male reproductive tract, specifically in the epididymis [1,128,129]. The expression of RNases 9 and 10 are directly and indirectly regulated by androgens or other testicular hormones [130]. Early work suggested that RNase 9 plays an important role in sperm maturation [131]. However, it was also found that the observed impairment in sperm motility in RNase 9-null mice was short-lived and thus did not affect their overall fertility [129,131]. Due to similarities in sequence and structure to the antimicrobial RNase A superfamily members, it is postulated that RNase 9 may play a role in host defense [132]. In support of this role, recombinant human RNase 9 peptide shows dose-dependent bactericidal activity against *E. coli* [66]. Single nucleotide polymorphisms (SNPs) in RNases 9 and 10 were shown to significantly affect interferon gamma secretion following rubella vaccination [65].

## 12. Other Immunomodulatory Ribonucleases

Onconase is a member of the Ribonuclease A Superfamily with strong cytotoxicity to cancer cells in vitro and in vivo that was first isolated from frog oocyte extracts [4,21,133,134]. In addition to its anti-tumor properties, onconase also displays remarkable antiviral activity and this may be due to upregulation of factors that suppress viral genome replication [135]. Onconase is currently in clinical trial for treatment of genital warts (clinical trial identifier: NCT02535104 at https://clinicaltrials.gov/). The mechanism for onconase-mediated cytotoxicity involves binding to anionic components of the extracellular membrane, cytosolic internalization, and degradation of tRNA leading to cytostasis and subsequent apoptosis [136]. This member of the RNase A Superfamily is a highly promising therapeutic agent in the treatment of various types of cancer.

Ribonucleases of the T2 family are found in the genomes of protozoans, plants, bacteria, animals, and viruses. A broad range of biological roles for these ribonucleases have been suggested. RNase T2 shares some common features with the RNase A Superfamily members. Like RNase 1, RNase T2 acts as an extracellular RNA scavenger [137]. Similar to RNase 2, RNase T2 serves as an alarmin, secreted by damaged tissues and signaling for immune response [138]. It also exhibits chemotactic properties, recruiting macrophages and initiating an immune response in vitro and in vivo [139]. Recently, RNase T2 has been implicated in neurodevelopment, modulation of the host immune response, and cancer [140,141,142,143]. The structure and function of RNase T2 family members and their biological roles has been reviewed [137].

## 13. Clinical Implications in Health and Disease

As highlighted above, RNase A peptides possess immunomodulatory and host defense properties that promote health and survival (Table 1 and Figure 4). Following tissue damage, RNases are expressed as alarmins. Their secretion or tissue deposition at sites of injury and inflammation promote tissue repair and remodeling. In doing so, they target and remove damaged cells and their released byproducts. To further promote tissue remodeling, RNase peptides function as chemokines and cytokines—acting as anti-inflammatories and attracting immune cells (i.e., macrophages or dendritic cells) to the source of injury. As described above, other immunomodulatory roles include removal of eRNA, activation of TLR, and binding nucleic acids [21]. To facilitate pathogen clearance, RNases are constitutively expressed to prevent pathogen attachment or invasion into epithelial cells. RNases can also be induced or secreted to directly participate in killing invading pathogens. After prolonged periods of infection, RNase expression can be down-regulated [9,55,116,144,145].

Given their expression during inflammatory states and infection, preclinical and clinical studies are evaluating the utility of RNase peptides as biomarkers for lower respiratory tract disease, inflammatory skin lesions, urinary tract infection, cancers, and sepsis [25,33,61,74,138,146,147,148]. In addition, a significant amount of research has been performed by multiple research teams to develop RNase peptides as novel therapies—including anti-inflammatories, antimicrobials, neuroprotection, tissue remodeling, angiogenesis, and chemotherapies [35,136,139,149,150,151].

## 14. Conclusions

This review highlights the contributions of the RNase A Superfamily to host defense and immune modulation. While many of the peptides in this family have similar functions, a greater understanding of their physiologic roles is needed. As these roles are elucidated, new methods and strategies will emerge to develop RNase A peptides as novel biomarkers and therapies of common human infections and inflammatory diseases described above. Given the clinical impact of these diseases, identifying mechanisms to develop RNase A Superfamily members as new therapies may have significant benefits to public health.

## Figures and Tables

**Table 1:** Summary of innate immune functions of Ribonuclease A Superfamily members.

Ribonuclease	Expression ^a^	Immunomodulatory Functions and Activities
RNase 1	pancreas, brain, gastrointestinal tract, endothelium, male reproductive tract, placenta, adipose tissue	Degrades extracellular RNA [21,22,23], stimulates dendritic cell maturation [24,25], antiviral in context of HIV-1 infection [21,26]
RNase 2 (EDN)	eosinophils, macrophages, dendritic cells, liver, lung, spleen	Alarmin [24,27,28], broad spectrum antiviral [16,18,27], dendritic cell chemotaxis and activation [24,28]
RNase 3 (ECP)	neutrophils, eosinophils	Antimicrobial [11], promotes bacterial cell agglutination [29,30], tissue remodeling [31,32], chemotaxis of fibroblasts [32,33,34]
RNase 4	liver, lungs, monocytes, mast cells	Angiogenesis [35,36], antiviral [37], sequence specific RNA cleavage [38,39], antimicrobial [40,41], neuroprotective [35,36]
RNase 5 (angiogenin)	liver, mast cells, endothelium	Angiogenesis [42,43], hematopoiesis [44], inhibits neutrophil degranulation [45], modulates inflammatory response [46], possible antimicrobial activity [43,47,48,49], cleaves tRNA and upregulates rRNA transcription [50,51,52], induces tiRNA fragments with stress [53], neuroprotective [54], tissue remodeling [48]
RNase 6	monocytes, neutrophils, lung, spleen	Antimicrobial [55,56], promotes bacterial cell agglutination [55], antiviral [57]
RNase 7	skin, urinary tract, respiratory tract	Alarmin [58], antimicrobial [15,59,60,61], promotes inflammation and chemotaxis of immune cells [48,58,62], tissue remodeling [48,63]
RNase 8	placenta, lung, liver, testes	Possible antimicrobial activity [17,63], possible role in amniotic cavity sterility [10,64]
RNase 9–13	male reproductive tract	Modulates cytokine secretion [65], antimicrobial [66]

^a^ Expression data was condensed from Human Protein Atlas (http://www.proteinatlas.org) and Uniprot (http://www.uniprot.org). EDN: Eosinophil Derived Neurotoxin; ECP: Eosinophil Cationic Peptide.
